# Oroxylin A inhibits glycolysis-dependent proliferation of human breast cancer via promoting SIRT3-mediated SOD2 transcription and HIF1*α* destabilization

**DOI:** 10.1038/cddis.2015.86

**Published:** 2015-04-09

**Authors:** L Wei, Y Zhou, C Qiao, T Ni, Z Li, Q You, Q Guo, N Lu

**Affiliations:** 1 State Key Laboratory of Natural Medicines, Jiangsu Key Laboratory of Carcinogenesis and Intervention, China Pharmaceutical University, 24 Tongjiaxiang, Nanjing 210009, People's Republic of China; 2 JiangSu Key Laboratory of Drug Design and Optimization, China Pharmaceutical University, 24 Tongjiaxiang, Nanjing 210009, People's Republic of China

## Abstract

Alterations of cellular metabolism play a central role in the development and progression of cancer. Oroxylin A, an active flavonoid of a Chinese traditional medicinal plant, was previously shown to modulate glycolysis in cancer cells. However, the mechanism by which oroxylin A regulates glycolysis is still not well defined. Here, we show that oroxylin A inhibits glycolysis in breast cancer cells via the Sirtuin 3 (SIRT3)-mediated destabilization of hypoxia-inducible factor 1*α* (HIF1*α*), which controls glycolytic gene expression. Oroxylin A promotes superoxide dismutase (SOD2) gene expression through SIRT3-regulated DNA-binding activity of FOXO3a and increases the activity of SOD2 by promoting SIRT3-mediated deacetylation. *In vivo*, oroxylin A inhibits the growth of transplanted human breast tumors associated with glycolytic suppression. These data indicate that oroxylin A inhibits glycolysis-dependent proliferation of breast cancer cells, through the suppression of HIF1*α* stabilization via SIRT3 activation, providing preclinical information for the cancer therapies of SIRT3 stimulation.

In contrast to normal cells, which rely primarily on mitochondrial oxidative phosphorylation to generate the energy needed for cellular processes, most malignant cells instead rely on aerobic glycolysis, a phenomenon termed 'the Warburg effect'. The Warburg effect has emerged as a metabolic hallmark of many cancers;^1^ however, the underlying mechanisms remain unclear. As limitations in tumor vascularization result in periods of intermittent hypoxia that force cells to rely on glycolysis,^2^ accumulating evidence suggests that aerobic glycolysis produces more than 50% of the cellular energy,^3^ and provides a survival advantage for tumor cells.

Metabolic reprogramming in cancer cells is regulated by several oncogenic cues, such as hypoxia-inducible factor 1*α* (HIF1*α*), which has been implicated in regulating many of the genes responsible for the metabolic switch.^4^ HIF1*α* stimulates glycolytic energy production by activating genes involved in extracellular glucose import, such as glucose transporter 1 (GLUT1) and hexokinase II (HKII).^5^ It downregulates oxidative phosphorylation within the mitochondria by transcriptional activation of genes such as pyruvate dehydrogenase kinase 1 (PDK1).^6^

Sirtuins is a conserved family of NAD-dependent ADP-ribosyltransferases and/or protein deacetylases involved in metabolism, stress responses, and longevity.^7^ Sirtuin 3 (SIRT3) is a key regulator of mitochondrial functions that result in the deacetylation of many enzymes involved in central metabolism, regulating numerous metabolic pathways.^8^ Recent studies showed that SIRT3 acts as a tumor suppressor through regulating mitochondrial integrity and metabolism in breast cancer.^9, 10^ Especially, increased levels of SIRT3 transcription were associated with node-positive breast cancer in clinical reports.^11^ SIRT3 also functions as a tumor suppressor by targeting the mitochondrial enzyme manganese superoxide dismutase (MnSOD), decreasing reactive oxygen species (ROS) production and maintaining genomic stability.^12^ Under hypoxic condition, HIF1*α* is stabilized by inhibition of the prolyl hydroxylases (PHDs) through the generation of ROS.^13^ Therefore, SIRT3 can control the stabilization of HIF1*α* by regulating mitochondrial ROS levels.^14^

Although low level of ROS contributes to cell signaling and cell proliferation, an excess of ROS, because of its highly reactive nature, causes damages to cellular constituents, including proteins, lipids and, in particular, DNA.^15^ Mitochondrial superoxide dismutase 2 (SOD2) is an important antioxidant enzyme that reduces the superoxide anion to regulate cellular redox homeostasis.^16^ Mitochondrial SIRT3 can interact with the forkhead box O3a (FOXO3a) proteins in mitochondria and activate the FOXO3a-dependent antioxidant-encoding gene SOD2.^17^ Moreover, SOD2 is a substrate of SIRT3 in mitochondria, and the binding of SIRT3 with SOD2 results in the deacetylation and activation of SOD2.^18^

Oroxylin A (OA), an active component of a Chinese traditional medicinal plant *Scutellaria baicalensis Georgi*, was previously shown to modulate glycolysis in cancer cells. Not only did OA inhibit glucose metabolism via two p53-related metabolic regulators, TIGAR and SCO2, but it also induced dissociation of HKII from the mitochondria through SIRT3.^19, 20^ In the present study, we found that OA suppressed cellular oxidative stress through SIRT3 under hypoxia, resulting in the destabilization of HIF1*α*. This is a novel anticancer mechanism of OA to inhibit cell growth and glycolysis in breast cancer.

## Results

### OA inhibits glycolysis in breast cancer cells by upregulating SIRT3 and SIRT3-mediated destabilization of HIF1*α*

**Figure 1:**
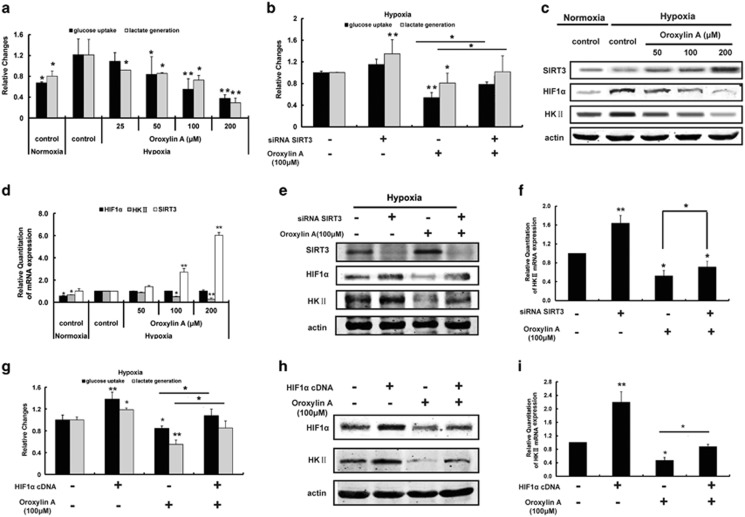
OA inhibits glycolysis by upregulating SIRT3 and SIRT3-mediated destabilization of HIF1*α*. (**a**) MDA-MB-231 cells were treated with OA under conditions of hypoxia or normoxia for 10 h. Glucose uptake was measured using the Amplex Red assay, and production of lactic acid was assayed with the Lactic Acid production Detection kit. (**b**) SIRT3-deficient MDA-MB-231 cells were incubated with 100 *μ*M OA for 10 h under hypoxia. Glucose uptake and production of lactic acid were assayed as above. (**c**, **d**) The protein expressions (**c**) and mRNA expressions (**d**) of SIRT3, HIF1*α* and HK II was detected by immunoblotting and quantitative RT-PCR, respectively, in cells treated with OA for 10 h under hypoxia. (**e**, **f**) SIRT3-deficient cells were treated with/without OA for 10 h under hypoxia. The protein expressions of SIRT3, HIF1*α* and HK II (**e**) and the mRNA expression of HK II (**f**) were detected. (**g**–**i**) HIF1*α* overexpressed MDA-MB-231 cells were then incubated with 100 *μ*M OA for 10 h under conditions of hypoxia. Glucose uptake and production of lactic acid (**g**), the protein expression (**h**) and the mRNA expression (**i**) of HK II were assayed. Bars, S.D.; *P<0.05 or **P<0.01 *versus* untreated controls in hypoxia

As shown in Figure 1a, under hypoxic condition, the glucose uptake and lactate production were both increased compared with that under normoxia condition. The glucose uptake and lactate production in breast cancer MDA-MB-231 cells were inhibited by OA in a concentration-dependent manner (Figure 1a). Silencing of SIRT3 expression by a specific siRNA reversed the OA-induced inhibition of glucose uptake and lactate generation (Figure 1b), indicating that OA inhibited glycolysis via SIRT3 in MDA-MB-231 cells under hypoxic condition. Moreover, OA upregulated SIRT3 under hypoxia conditions at both protein and mRNA levels (Figures 1c and d). siRNA-mediated silencing of SIRT3 reversed the downregulated protein and mRNA levels of the HKII induced by OA (Figures 1e and f). HK II, a target gene of HIF1*α*, is critical for increased glucose uptake and catabolism via aerobic glycolysis.^21, 22^ Under hypoxia condition, OA induced more expression of HKII following SIRT3 knockdown, suggesting the important role of SIRT3 in regulating the metabolic response to hypoxia (Figure 1e). Other than MDA-MB-231 cells, OA also inhibited glycolysis of breast cancer MCF-7 cells under hypoxia via increasing SIRT3, which was increased in the mRNA level as well as in protein level (Supplementary Figure 1A,1B, 1C and 1E).

As HIF1*α* stimulates the expression of glycolytic enzymes and decreases reliance on mitochondrial oxidative phosphorylation in tumor cells,^23^ we speculated that OA may regulate glycolysis via SIRT3 through a process involving HIF1*α*. To test this idea, we first investigated whether SIRT3 modulated HIF1*α* stability. OA decreased the protein expression of HIF1*α* in MDA-MB-231 cells and MCF-7 cells (Figure 1c,Supplementary Figure 1E). SIRT3 siRNA reversed the OA-induced HIF1*α* destabilization (Figure 1e,Supplementary Figure 1F), suggesting that OA destabilizes HIF1*α* associated with SIRT3. Importantly, under hypoxic condition, the inhibition of glucose uptake, lactate production (Figure 1g), and the regulation of HK II (Figures 1g–i) induced by OA were abolished by overexpression of HIF1*α*. These findings indicated that OA inhibited glycolysis by repressing the activity of HIF1*α*.

### OA downregulates HIF1*α* through increasing PDH activity by SIRT3

**Figure 2:**
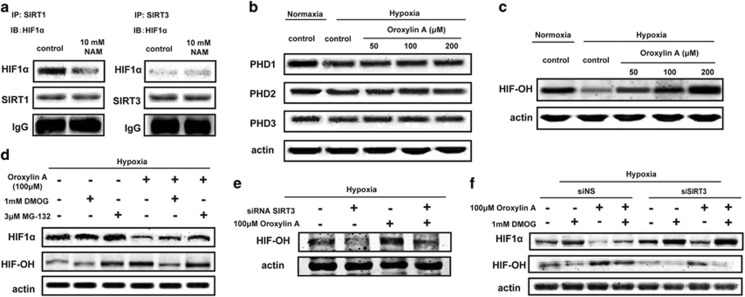
OA destabilized HIF1*α* by influencing SIRT3-modulated activity of PDH. (**a**) MDA-MB-231 cells were treated with NAM (10 mM) for 10 h under hypoxic conditions. HIF1*α* was immunoprecipitated using SIRT3 or SIRT1 antibodies. Western blot assays were performed for HIF1*α*, SIRT1 and SIRT3. (**b**) For 10 h OA treatment under hypoxia, the expression of PHD was detected by immunoblotting. (**c**) MDA-MB-231 cells were treated with OA for 10 h under hypoxic or normoxia conditions. The expression of hydroxylated HIF1*α* (HIF-OH) was detected by immunoblotting. (**d**) MDA-MB-231 cells treated with or without 100 *μ*M OA or 3 *μ*M MG-132 or 1 mM DMOG for 10 h as indicated were immunoblotted against antibodies specific to HIF-OH, or total HIF1*α*. (**e**) SIRT3-deficient MDA-MB-231 cells were incubated with 100 *μ*M OA for 10 h under hypoxia. HIF-OH was detected by immunoblotting. (**f**) SIRT3-deficient MDA-MB-231 cells were incubated with or without 100 *μ*M OA or 1 mM DMOG for 10 h under hypoxia. Hydroxylated HIF1*α* (HIF-OH) and total HIF1*α* were detected by immunoblotting

To further study the effect of OA on HIF1*α*, the expressions of HIF*α* at mRNA and protein levels were examined. As no notable difference of HIF*α* mRNA level was observed (Figure 1d and Supplementary Figure 1C), we hypothesized that the regulation of HIF1*α* may occur at the posttranslational level. It is reported that SIRT1 binds HIF1*α* and regulates its activity through direct deacetylation.^24^ To test whether OA regulated HIF1*α* via SIRT3 through a similar mechanism, we checked the interaction of SIRT1 or SIRT3 with HIF1*α*. As shown in Figure 2a, under hypoxia condition, SIRT1 interacted with HIF1*α*, but SIRT3 did not; and the deacetylase inhibitor nicotinamide (NAM) inhibited the binding of HIF1*α* with SIRT1, suggesting that HIF1*α* cannot be deacetylated by SIRT3.

Usually, HIF-1*α* is controlled by cellular oxygen concentrations via PHDs and the von Hippel–Lindau complex, and is easily degraded in normoxia.^25^ We next tested whether OA influenced SIRT3-modulated HIF1*α* stability by modulating PHD activity. OA had little influences on the protein expressions of PHDs (PHD1, PHD2 and PHD3) (Figure 2b). Then, PHD activity was measured by the level of HIF1*α* hydroxylation.^26^ As shown in Figure 2c, the levels of hydroxylated HIF1*α* (OH-HIF) were increased in response to OA treatment for 10 h under hypoxia. The activity of PHD in hypoxia was then assessed in control and OA-treated MDA-MB-231 cells pre-treated with the proteasomal inhibitor MG-132 (to prevent HIF1*α* from being degraded) or with dimethyloxalylglycine (DMOG; to inhibit PHDs). Although both DMOG and MG-132 attenuated the OA-induced destabilization of HIF1*α*, only DMOG reversed the OA-induced increase in the level of hydroxylated HIF1*α*, indicating that OA indeed regulated HIF1*α* stability through modulating PHD activity (Figure 2d). Moreover, the hydroxylation of HIF1*α* induced by OA was decreased by knocking down SIRT3 (Figure 2e). To confirm the role of SIRT3 in OA-modulated HIF1*α* stability via inactivating PHDs, we further examined the effects of DMOG on PHD/HIF *α* in MDA-MB-231 with different levels of SIRT3. As shown in Figure 2f, the response of SIRT3-deficient cells to DMOG was strengthened, underscoring the regulation of SIRT3 on the role of PHD. Blocking PHD activity by DMOG decreased the levels of hydroxylated HIF1*α*, thus increasing the stability of HIF1*α* in response to OA, which is stronger than that in SIRT3 knocked down cells. These results suggest that the increase in PHD activity induced by SIRT3 is the mechanism of HIF1*α* destabilization in OA-treated cells.

### OA regulates the activity of PHD through inhibiting mitochondrial ROS production

**Figure 3:**
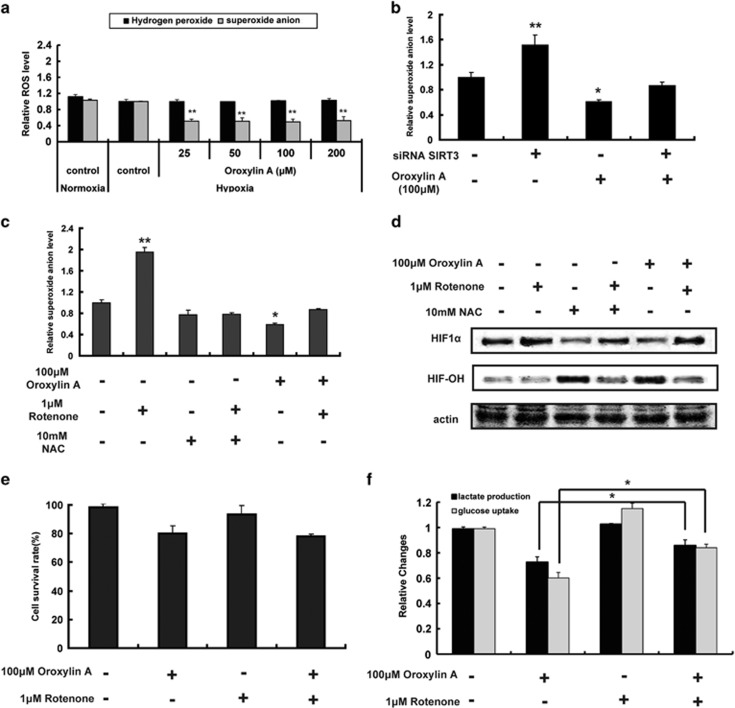
OA-induced activation of PDHs through the regulation of **·**O_2_^−^ levels by SIRT3. (**a**) MDA-MB-231 cells were treated with OA for 10 h under hypoxia. The levels of cellular superoxide anion (**·**O_2_^−^) and hydrogen peroxide (H_2_O_2_) were detected by FACSCalibur flow cytometry using the fluorescent dye dihydroethidium at Ex/Em of 300/610 nm or dichlorofluorescein-diacetate at Ex/Em of 488/525 nm, respectively. (**b**) SIRT3-deficient MDA-MB-231 cells were incubated with or without 100 *μ*M OA for 10 h under hypoxia, and **·**O_2_^−^ was detected. (**c**, **d**) Cells were treated with 100 *μ*M OA or 10 mM *N*-acetyl cysteine (antioxidant used as a positive control) in the presence of 1 *μ*M rotenone for 10 h under hypoxia. The levels of **·**O_2_^−^ (**c**), protein expression of HIF-OH and HIF1*α* (**d**) were detected. (**e**, **f**) Cells were treated with 100 *μ*M OA with/without 1 *μ*M rotenone for 10 h under hypoxia. Cell survival rate (**e**), glucose uptake and production of lactic acid (**f**) were detected, respectively. Bars, S.D.; *P<0.05 or **P<0.01 *versus* untreated control

Increased intracellular ROS levels triggered by hypoxia are known to inhibit PHDs and stabilize HIF1*α*.^27, 28^ Therefore, we hypothesized that the decrease in ROS levels induced by OA via SIRT3 would contribute to the increased PHD activity under hypoxic condition. Treatment with different concentrations of OA under hypoxic conditions decreased the level of superoxide anion (**·**O_2_^−^) but did not change the level of hydrogen peroxide (H_2_O_2_) (Figure 3a and Supplementary Figure 1G). The total ROS level was decreased. We further tested whether deletion of SIRT3 would attenuate the decrease in **·**O_2_^−^ level induced by OA. As a result, the level of **·**O_2_^−^ was significantly higher in SIRT3-deficient cells than untransfected MDA-MB-231 cells, and OA-triggered decrease in **·**O_2_^−^ levels was reversed by SIRT3 depletion (Figure 3b, Supplementary Figure 1H). Next, we treated cells with rotenone to determine whether the generation of **·**O_2_^−^ was involved in the effects of OA on HIF1*α* stability. Increased **·**O_2_^−^ levels were induced by rotenone without influence of cell growth (Figures 3c and e). As shown in Figure 3d, the increase in **·**O_2_^−^ level induced by rotenone blocked the OA-mediated activation of PHD, decreasing hydroxylated HIF1*α* and increasing HIF1*α* levels. Rotenone also blocked the effect of OA on the inhibition of glucose uptake and lactate production (Figure 3f).These findings indicate that the regulation of **·**O_2_^−^ levels by SIRT3 plays an important role in OA-induced activation of PDH, destabilization of HIF1*α*, and suppression of glycolysis.

### OA reduced ·O_2_^−^ level by the SIRT3-mediated upregulation of SOD2

**Figure 4:**
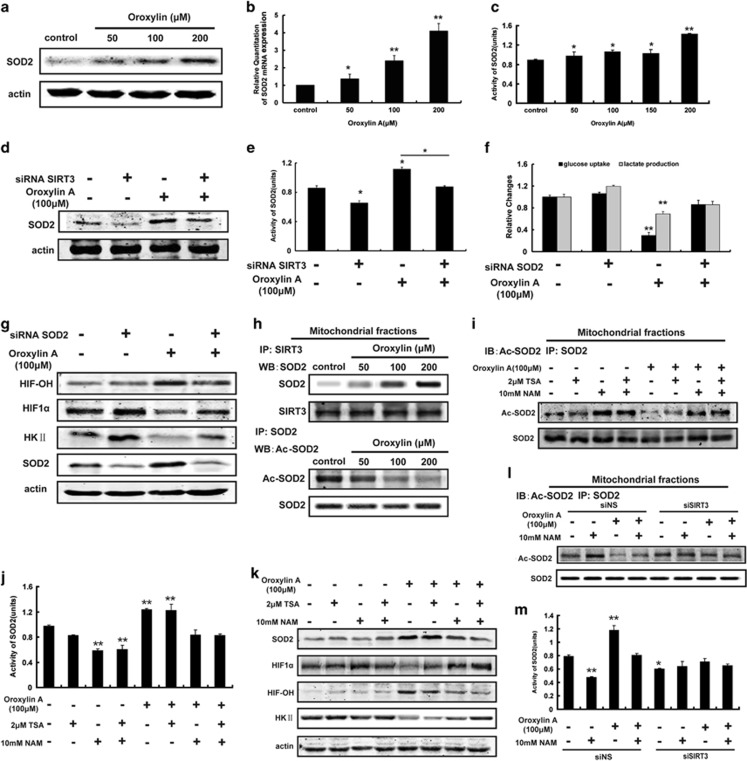
The regulations of OA on glycolysis and HIF1*α* were associated with increased SOD2 levels under hypoxia. (**a**–**c**) MDA-MB-231 cells were treated with OA for 10 h under hypoxia. The protein expression (**a**), mRNA expression (**b**) and activity (**c**) of SOD2 were detected by immunoblotting, Cu/Zn-SOD and Mn-SOD Assay Kit, and quantitative RT-PCR, respectively. (**d**, **e**) SIRT3-deficient MDA-MB-231 cells were incubated with or without 100 *μ*M OA for 10 h under hypoxia conditions. The protein expression (**d**) and activity (**e**) of SOD2 were detected as described above. (**f**, **g**) SOD2-deficient MDA-MB-231 cells were incubated with or without 100 *μ*M OA for 10 h under hypoxia. Glucose uptake and production of lactic acid (**f**), and protein expressions of HIF-OH, HIF1*α*, and HKII (**g**) were detected. (**h**) MDA-MB-231 cells were treated with OA for 10 h under hypoxia. SOD2 was immunoprecipitated using SIRT3 antibody, and acetylated-SOD2 (Ac-SOD2) was immunoprecipitated using SOD2 antibody in mitochondrial extracts. (**i**–**k**) MDA-MB-231 cells were treated with or without 100 *μ*M OA or 2 *μ*M TAS or 10 mM NAM for 10 h under hypoxia. (**i**) Ac-SOD2 was immunoprecipitated using SOD2 antibody in mitochondrial extracts. (**j**) The activity of SOD2 was detected. (**k**) The protein expressions of SOD2, HIF-OH, HIF1*α*, and HK II was detected by immunoblotting. (**l**, **m**) SIRT3-deficient MDA-MB-231 cells were incubated with or without 100 *μ*M OA or 10 mM NAM for 10 h under hypoxia. Ac-SOD2 was immunoprecipitated in mitochondrial extracts (**l**), and the activity of SOD2 (**m**) was assayed. Bars, S.D.; *P<0.05 or **P<0.01 *versus* untreated controls

Mitochondrial SOD (SOD2, also known as Mn-SOD) plays a crucial role in controlling the level of ROS by clearing **·**O_2_^−^.^29^ In breast cancer MDA-MB-231 cells and MCF-7 cells, the protein level, mRNA level (Figures 4a and b, Supplementary Figure 1C and 1E), and the activity of SOD2 (Figure 4c, Supplementary Figure 1I) were all increased in response to OA treatment under hypoxic conditions. Moreover, the downregulated level and activity of SOD2 by OA were attenuated by SIRT3 deficiency (Figures 4d and e, Supplementary Figuer 1D, 1F, and 1J). These findings indicated that the OA-induced upregulation of SOD2 level was associated with SIRT3 to some degree. Furthermore, OA-induced inhibition of glucose uptake, lactate production, and the expression of HK II were reversed by knocking down SOD2 (Figures 4f and g). Moreover, silencing of SOD2 enhanced the stability of HIF1*α* and decreased PDH activity (Figure 4g).

As calorie restriction leads to SIRT3-mediated deacetylation of MnSOD/SOD2 and the subsequent increase of its antioxidant activity,^30^ we further studied the relationship between the influence of OA on SOD2 activity and SIRT3. As shown in Figure 4h, OA promoted the binding of SOD2 and SIRT3 in mitochondria, and resulted in the decreased acetylation of SOD2. SIRT1 inhibitor NAM and trichostatin A (TSA, a selective inhibitor of histone deacetylase) inhibits most known deacetylases. Only NAM increased the deacetylation of SOD2 (Figure 4i), and decreased the activity of SOD2 significantly (Figure 4j). Both NAM and TSA reversed the destabilization of HIF1*α* and the downregulation of HK II induced by OA; and the effect of NAM was stronger than TSA (Figure 4k). However, only NAM blocked the OA-induced decrease of the acetylated SOD2 (Figure 4i) and the upregulated expression and activity of SOD2 (Figures 4j and k). Loss of SIRT3 diminished the effects of OA and NAM on the deacetylation of SOD2 (Figure 4l) and the activity of SOD2 (Figure 4m). These results suggest that the deacetylation of SOD2 induced by OA is mediated by SIRT3 and OA enhances SOD2 activity through the deacetylation of SIRT3.

### OA upregulates SOD2 through the interaction of SIRT3 with FOXO3a

**Figure 5:**
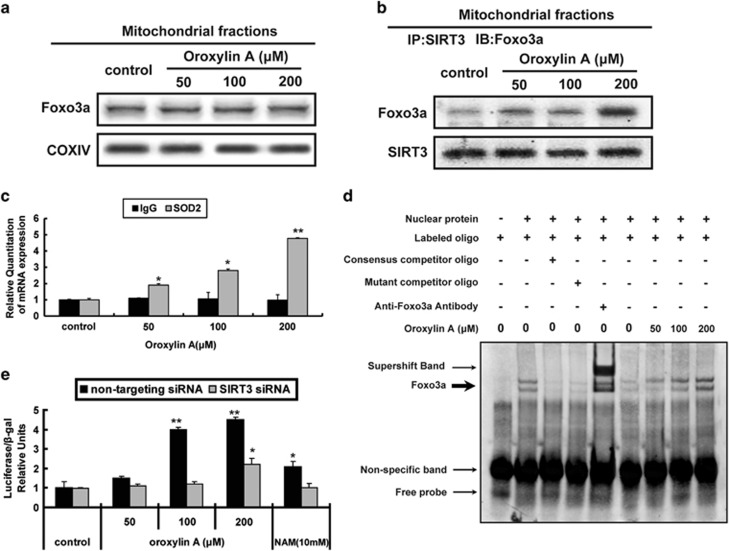
OA regulated the mRNA expression of SOD2 through the interaction of SIRT3 with FOXO3a in mitochondria. (**a**–**d**) MDA-MB-231 cells were treated with OA for 10 h under hypoxia conditions. (**a**) Mitochondria were isolated after treatment and subjected to western blot analysis for FOXO3a. (**b**) FOXO3a was immunoprecipitated using a SIRT3 antibody. (**c**) ChIP analysis of FOXO3a binding to the SOD2 promoter in MDA-MB-231 cells treated with OA. Cells were fixed with 1% formaldehyde to crosslink protein–DNA interactions, sonicated, and fixed cells were immunoprecipitated with anti-FOXO3a antibody. DNA was eluted and purified before analysis using specific primers and quantitative RT-PCR. (**d**) Nuclei were isolated after treatment and subjected to EMSA to assess the binding of FOXO3a to the SOD2 promoter in mitochondria. (**e**) SIRT3-deficient and normal MDA-MB-231 cells were treated with OA or 10 mM NAM for 10 h under hypoxia conditions. SOD2 promoter luciferase reporter plasmid (p3x-FOXO3a-luc) was transfected into MDA-MB-231 cells. Luciferase activity was normalized to Renilla activity and expressed as luciferase/Renilla relative units. Bars, S.D.; *P<0.05 or **P<0.01 *versus* untreated controls

It has been demonstrated that FOXO3a plays an important role in regulating the expression of SOD2.^31^ Moreover, SIRT3-mediated deacetylation of FOXO3a promotes its nuclear localization. Therefore, we investigated whether FOXO3a could interact with SIRT3 in mitochondria. As a result, FOXO3a localized in mitochondria and OA had little influence on the total protein level of FOXO3a (Figure 5a). However, OA promoted the interaction of FOXO3a with SIRT3 in the mitochondria (Figure 5b). We further investigated the role of FOXO3a in OA-induced SOD2 expression using chromatin immunoprecipitation (ChIP) and electrophoretic mobility shift assay (EMSA) assays. In ChIP assay, we found that OA promoted the binding of FOXO3a to the promoter of SOD2 (Figure 5c). The results of EMSA also showed that OA increased the binding of the exogenous consensus DNA oligonucleotide of SOD2 with FOXO3a (Figure 5d). Moreover, FOXO3a-luciferase reporter gene assay indicated that OA increased the transcriptional activity of Foxo3a, whereas NAM blocked the effects of OA (Figure 5e). When the cells were co-transfected with the p3x-FOXO3a-luc plasmid and SIRT3 siRNA, the regulative activities of OA and NAM were abolished (Figure 5f). Collectively, these findings indicate that OA enhances SOD2 expression by promoting SIRT3-modulated FOXO3a transcriptional activity.

### OA inhibits the growth of breast cancer cells through SIRT3-associated inhibition of glycolysis

**Figure 6:**
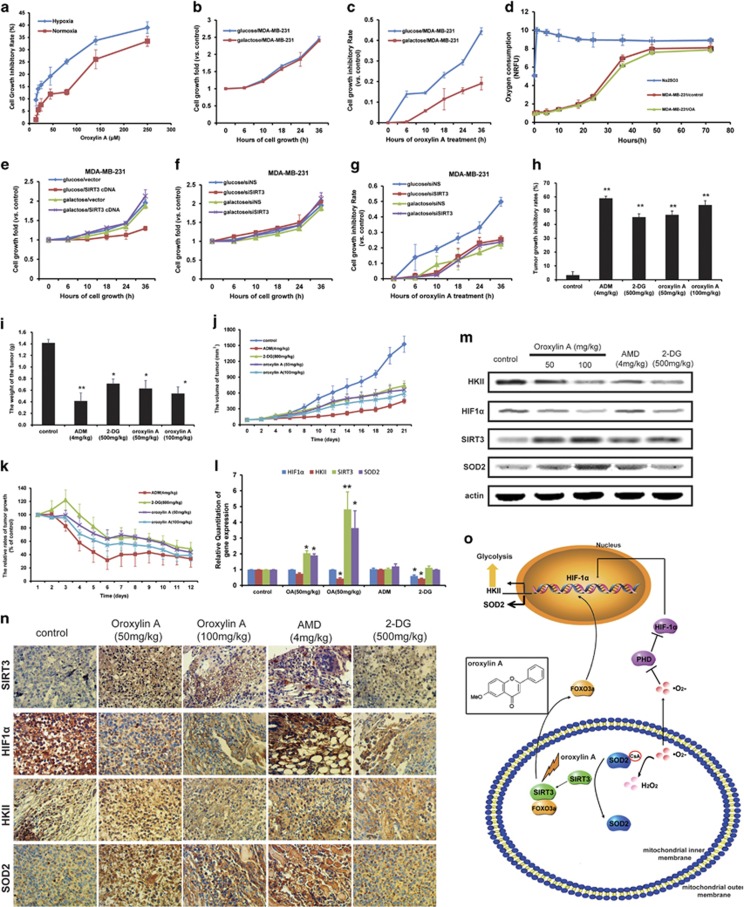
OA inhibited the growth of human breast cancer cells and transplanted human breast tumor. (**a**) Cells were treated with OA for 10 h under hypoxia or normoxia. The growth-inhibitory effect of OA on MDA-MB-231 cells. (**b**) Growth curves of MDA-MB-231 cells cultured in media containing glucose or galactose under normoxia. (**c**) Growth-inhibitory curves of MDA-MB-231 cells cultured in media containing glucose or galactose after treatment with 100 *μ*M OA for 36 h under normoxia. (**d**) Oxygen consumption of MDA-MB-231 cells. After treatment with 100 *μ*M OA for 72 h under normoxia, oxygen consumption was detected by BD Oxygen Biosensor System plate. (**e**) Growth curves of SIRT3-overexpressed MDA-MB-231 cells cultured in media containing glucose or galactose under normoxia. (**f**) Growth curves of SIRT3-deficient MDA-MB-231 cells cultured in media containing glucose or galactose under normoxia. (**g**) Growth-inhibitory curves of SIRT3-deficient MDA-MB-231 cells cultured in media containing glucose or galactose with 100 *μ*M OA for 36 h under normoxia. (**h**–**n**) Nude mice inoculated with MDA-MB-231 cells were treated with saline control, OA, 2-DG, and ADM. (**h**) The tumor-inhibitory rates were calculated. The tumor weight (**i**) and tumor volume (**j**) were measured. The relative rates of tumor growth (**k**) were calculated. (**l**) Quantitative RT-PCR on RNA isolated from xenograft tumors. (**m**) Protein expressions in breast tumor tissue were assessed by immunohistochemistry. (**n**) The tumor tissue protein expressions of xenograft tumors were assayed by immunoblotting. (**o**) Schematic diagram of OA inhibition of glycolysis and growth of breast cancer cells through SIRT3-mediated regulation of HIF1*α*. Bars, S.D., **P<0.01 *versus* untreated controls

OA inhibited the growth of MDA-MB-231 cells under hypoxia as well as normoxia (Figure 6a), but exhibited little cytotoxicity on cell survival (Supplementary Figure 6). To test whether the aerobic metabolism of glucose was essential to cell growth, we employed the media containing galactose instead of glucose under normoxia, which could reduce the glycolytic flux and force the growing of cells relying on mitochondrial oxidative phosphorylation.^32^ Under these conditions, the growth of breast cancer cells was not affected (Figure 6b), whereas the growth-inhibitory effects of OA were attenuated by the replacement of glucose with galactose (Figure 6c). Furthermore, OA did not regulate the oxygen consumption in cells (Figure 6d). These results suggest that OA inhibits the growth of cancer cells by regulating aerobic glycolysis (Warburg effects) instead of oxidative phosphorylation.

Then, we explored the importance of SIRT3 in glycolysis-dependent growth. Overexpression of SIRT3 decreased the growth rate of cells cultured in the presence of glucose, not in the presence of galactose (Figure 6e), indicating that SIRT3 involves in glucose catabolism-regulated cell growth; whereas the deletion of SIRT3 did not influence the growth of cells cultured in the presence of glucose, or in the presence of galactose (Figure 6f). As shown in Figure 6g, although the reduction in the rate of glycolysis induced by replacing glucose with galactose attenuated the growth-inhibitory activity of OA, the deletion of SIRT3 abolished the activity of OA. These results suggest that OA inhibits the growth of breast cancer cells through the inhibition of SIRT3-associated glycolysis.

Furthermore, we performed xenograft experiment with MDA-MB-231 cells. As shown in Figure 6h, the inhibitory effect of 100 mg/kg OA on tumor growth was stronger than that of 50 mg/kg OA or 500 mg/kg 2-deoxyglucose (2-DG) and weaker than that of 2 mg/kg Adriamycin (ADM). Tumor volume and tumor mass were lower in OA-, 2-DG-, or ADM-treated mice than in the control group on the same measurement day (Figures 6i–k). Because hypoxia has been shown to affect the center of solid tumors,^33^ center tissue extracted from the tumor samples of nude mice were used for gene and protein expression assay. In the OA-treated group, increased SIRT3 and decreased level of HIF1*α*; as well as upregulated SOD2 and downregulated HKII levels were shown (Figures 6l, n, and o). Moreover, OA also inhibited the tumor growth of the nude mice inoculated with MCF-7 cells (Supplementary Figure 1K). The influences of OA on the protein expressions of HIF1*α*, SIRT3, and SOD2 in tumor tissue of the nude mice inoculated with MCF-7 cells were consistent with that inoculated with MDA-MB-231 cells (Supplementary Figure 1L).

Collectively, all these results suggest that OA inhibits tumor growth through the regulation of SIRT3-mediated HIF1*α* destabilization.

## Discussion

In the present study, we show that OA inhibits glycolysis in breast cancer cells through SIRT3-mediated HIF1*α* destabilization, which has important implications for treatment of cancers. OA, a natural product from *Scutellaria*, can upregulate SIRT3 expression and inhibit glycolysis in breast cancer cells under hypoxia through enhancing SIRT3-mediated stabilization and activation of HIF1*α* (Figures 1 and 2). Decreased **·**O_2_^−^ levels in OA-treated cells increase the activity of PHD and destabilize HIF1*α* (Figure 3). Further investigation reveals the key role of the mitochondrial anti-oxidative enzyme SOD2 in the regulation of **·**O_2_^−^ levels in OA-treated cells (Figure 4). OA not only enhances the activity of SOD2 via promoting SIRT3-mediated deacetylation (Figure 4), but also increases SOD2 expression by increasing the transcriptional activity of FOXO3a (Figure 5). Moreover, OA inhibits glycolysis-dependent growth of breast cancer cells, rather than oxidative phosphorylation (Figure 6). These results highlight the potential importance of OA in SIRT3-mediated metabolic reprogramming in human breast cancers. *In vivo*, OA also suppresses tumor growth through destabilizing HIF1*α* and upregulating SIRT3 (Figure 6). Taken together, these data provide a mechanism by which OA inhibits glycolytic metabolism by promoting SIRT3-mediated decrease of HIF1*α* stability and activity (Figure 6o).

A clear understanding of how individual sirtuins function in different cancer types is important for assessing their potential as therapeutic targets.^34^ The involvement of sirtuins in tumorigenesis has promoted research into the effect of sirtuin inhibitors on different cancer cell lines.^35^ However, a recent study by Finley *et al.*^14^ shows that SIRT3 functions as a tumor suppressor, and loss of SIRT3 promotes tumorigenesis by altering global cellular metabolism. SIRT3 could suppress HIF1*α* and tumor growth by inhibiting mitochondrial ROS production.^36^ Our findings show that OA is an effective activator of SIRT3. Through promoting SIRT3-modulated transcription and activity of SOD2, OA destabilizes HIF1*α*, decreases the transcription of HIF1*α*-targeted gene HKII, and suppresses glycolysis. Furthermore, the nuclear translocation of HIF1*α* is promoted by OA as well (Supplementary Figure 4).

High levels of ROS results in DNA damage as well as damage to proteins and lipids, thereby promoting cell death, senescence, or aging.^15^ However, at lower levels, electron transport chain-generated ROS can promote cell division and growth, and modulate MAP kinase signaling cascades through the regulation of phosphatases and transcription factors.^15^ OA is a flavonoid that exhibits multiple pharmacological activities, including antioxidative, anti-inflammatory, anti-viral, and anti-tumor properties. Previous studies have shown that OA induces apoptosis by promoting the accumulation of ROS,^37, 38^ demonstrating the long-term efficacy of OA. In the present study, treatment with OA for 10 h under hypoxia decreased ROS production (Figure 3a), showing the short-term efficacy of OA. OA treatment for 36 h under hypoxia results in an increase in ROS levels (Supplementary Figure 2A) and the decrease of SOD2 level (Supplementary Figure 2B). In addition, the mRNA level of HIF1*α* as well as its protein level were both decreased upon OA treatment for 36 h under hypoxia (Supplementary Figure 2C). Moreover, the protein level of PDH2 was increased upon OA treatment for 36 h, instead PDH1 and PHD3 were not (Supplementary Figure 2D). Although the reason for the reversal pattern of ROS generation caused by OA is not clear, the time-dependent effects of OA are meaningful for its protective effect on normal tissue and therapeutic effect on malignant tumor tissue. Our previous work reported the protective effect of OA on normal hepatic cells.^39^ And we find that L02 cells have relatively lower intrinsic ROS levels than HepG2 cells (Supplementary Figure 3A), which might be responsible for the appearance that OA preferentially kills cancer cells than normal cells (Supplementary Figure 3B). In addition, normal liver cells have lower glycolytic activity than hepatoma cells, showing lower levels of glucose uptake, lactate production, and expressions/activities of glycolytic enzymes, as well as lower HIF1*α* level (Supplementary Figures 3C and 3E). Likewise, OA has less influence on the glycolysis of L02 cells than HepG2 cells. On the basis of all above, our results suggest that the Warburg effect is related to the high level of cellular ROS and HIF-1 in tumor cells, which is a potential target for cancer therapy.

Recent studies have shed light on the mechanism by which SIRT3 regulates cellular ROS level.^9, 40^ SIRT3 directly targets isocitrate dehydrogenase 2 (IDH2), affecting cellular redox status and SOD2-mediated scavenging of mitochondrial ROS.^30, 41, 42^ Additionally, several groups have provided evidence that SIRT3 promotes the transcription of SOD2 through the activation of FOXO3a.^17, 31^ In the present study, OA upregulates SOD2 mRNA expression through promoting SIRT3-regulated DNA-binding activity of FOXO3a. These results raise questions regarding the role of SIRT3 in mitochondria, as its effects on mitochondria result in its regulation of FOXO3a in the cytosol/nucleus. We find that OA promotes the nuclear translocation of Foxo3a (Supplementary Figure 5). The study by Prof. Gupta MP *et al.*^17^ demonstrated that SIRT3 was capable of promoting the nuclear localization of Foxo3a and enhancing the transcription of Foxo3a-dependent genes, which explained the principal effects of SIRT3 on FOXO3a.

SIRT3 regulates most of the cancer hallmark processes, suggesting SIRT3 can be considered as a novel potential therapeutic target for the treatment of cancer.^34^ Interestingly, the dichotomy of SIRT3's role in different cancer cell types is also highlighted.^34^ SIRT3 has been reported to act as a tumor promoter in fibrosarcoma, cervical cancer, and bladder cancer by protecting these tumors from stress-mediated cell death.^17, 43^ On the other hand, studies have shown that SIRT3 suppresses tumorigenesis and induces cell death in colorectal carcinoma, osteosarcoma, leukemia, ovarian cancer, and breast cancer.^9, 36, 44^ Even in normal cells such as neurons, the role of SIRT3 remains controversial.^45^ The discrepant roles of SIRT3 in cancer make its development as a potential target for cancer therapy confusing. Our results indicate that OA is an effective activator/regulator of SIRT3 in breast cancer, providing more extent information for evaluating the role of SIRT3 for cancer therapeutic intervention.

## Materials and Methods

### Reagents

OA (C_16_H_12_O_5_) was isolated from the root of *Scutellaria baicalensis* according to previously reported protocols.^46^ It was dissolved in DMSO as a stock solution and stored at −20 °C and diluted with medium before each experiment. The final concentration of DMSO did not exceed 0.1% in the study. DMOG, *N*-acetyl cysteine, 2-DG, galactose, and retonone were purchased from Sigma-Aldrich (St. Louis, MO, USA) and prepared to 10^−1^ M stock solutions following the instruction. Carbobenzoxy-Leu-Leu-leucinal (MG132), TSA, and NAM were purchased from Beyotime (Beyotime Institute of BioTechnology, Haimen, China). ADM was used as the reference drug, and was purchased from Sigma-Aldrich and dissolved in 0.9% NaCl before use.

### Cell culture

The human breast cancer cell line MDA-MB-231 was purchased from Cell Bank of Shanghai Institute of Biochemistry & Cell Biology, Chinese Academy of Sciences (Shanghai, China). MDA-MB-231 cells were cultured in RPMI-1640 (GIBCO, Invitrogen Corporation, Carlsbad, CA, USA) supplemented with 10% heat-inactivated FBS (Sijiqing, Hangzhou, China), 100 U/ml penicillin G, and 100 *μ*g/ml streptomycin at 37 °C, 95% relative humidity, and 5% CO_2_ with 20% oxygen (normoxia), or 1% oxygen (hypoxia). Galactose media was prepared as below: RPIM-1640 deprived of glucose (Invitrogen 11879-020) supplemented with 10 mM galactose, 5 mM HEPES, 10% FBS, 1 mM sodium pyruvate, and pen-strep as above.

### Cell growth assay

The cell growth was measured by colorimetric MTT assay. The logarithmic cells were detached to prepare 1.0 × 10^5^ /ml cell suspension, partitioned into 6-well plates at 1 ml per well for 24 h at 37 °C, and then cells were treated. After incubation, 5 mg/ml MTT (3-(4,5-dimethylthiazol-2-yl)-2,5-diph-enyltetrazolium bromide; Sigma-Aldrich) solution (450 *μ*l per well) was added and cultured for 4 h. Then, the supernatant was discarded and DMSO was added (2 ml per well). The suspension was placed on micro-vibrator for 5 min and the absorbance (A) was measured at 570 nm by the Universal Microplate Reader (EL800, BIO-TEK Instruments, Inc., Winooski, VT, USA). The cell survival rate was calculated by the following formula:


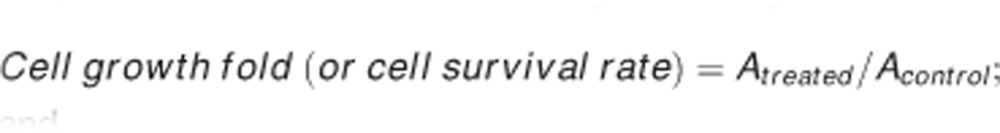


and


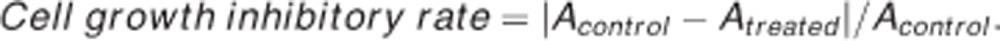


where *A*_*treated*_ and *A*_*control*_ were the average absorbance of three parallel experiments from the treated and blank control groups, respectively. The results were presented as mean±S.D.

### Measurements of lactate generation

Cells were seeded at a density of 2 × 10^5^ cells per well in 6-well cell culture plate. After cells were treated with OA for 10 h under hypoxia, culture media and cells were collected. Lactate generation was assayed following the manufacturer's instructions of the Lactic Acid production Detection kit (KeyGen, Nanjing, China). The absorbance was determined by Varioskan multimode microplate spectrophotometer (Thermo, Waltham, MA, USA) at 570 nm, and normalized as follows: OD_normalized_=OD_measured_ / living cell number_treated_ × living cell number_control_. The living cells were counted by trypan blue staining of collected cells. Then, the amount of lactate generation was calculated as follows: lactate generation (mM)=3 × (OD_sample normalized_ −OD_blank_)/(OD_standard_ −OD_blank_).

### Measurements of glucose uptake

Cells were seeded at a density of 2 × 10^5^ cells per well in 6-well cell culture plate. After cells were treated with OA for 10 h, culture media were collected and diluted 1 : 4000 in water. Glucose in the medium was quantitated via an Amplex Red Glucose/ Glucose Oxidase Kit (Invitrogen, Eugene, OR, USA) using a standard curve prepared with serial dilutions of RPMI 1640 (11 mmol/l glucose) into glucose-free RPMI 1640. Fluorescence was read using a Varioskan multimode microplate spectrophotometer (Thermo) at Ex./Em.=530 nm/590 nm, and normalized as the method in the section 'Measurements of lactate generation'. The concentration of glucose uptake in each sample was then calculated. Glucose uptake was determined by subtracting the amount of glucose in each sample from the total amount of glucose in the media without cells (11 mmol/l glucose).

### Measurements of oxygen consumption

Cells were seeded at a density of 0.2 × 10^6^ cells per well in a 96-well BD Oxygen Biosensor System plate (BD Biosciences, San Jose, CA, USA), and culture medium was added until a final volume of 200 *μ*l per well was attained. After drug treatments, the plates were maintained at 37 °C in a humidified incubator with 5% CO_2_. Plates were scanned in a temperature-controlled (37 °C) plate reader (Thermo) with an excitation wavelength of 485 nm and an emission wavelength of 630 nm at predetermined times for a total of 72 h. The fluorescence traces in each well were normalized according to the signal in the air-saturated buffer. Slopes of fluorescence signal were calculated in the dynamic range of measurements to compare the respiratory rates of samples. Normalized relative fluorescence unit represents the oxygen consumption.

### Measurement of hydrogen peroxide and superoxide anion levels

Cells were harvested and stained with H_2_O_2_-sensitive dye (5 *μ*M), 2',7'-dichlorofluorescein-diacetate (Beyotime Institute of BioTechnology) and **·**O_2_^−^-sensitive dye dihydroethidium (Beyotime Institute of BioTechnology). The fluorescence intensity was quantified by FACSCalibur flow cytometry (Becton Dickinson) at Ex./Em.=488 nm / 525 nm for H_2_O_2_ detection and Ex/Em=300 nm/610 nm for **·**O_2_^−^ detection, respectively.

### Preparation of mitochondrial extracts

After incubation with OA for 10 h under hypoxia, the fractionation of the mitochondrial protein was extracted according to Mitochondria/Cytosol Fractionation Kit (Biovision, Milpitas, CA, USA) instruction. Briefly, 5 × 10^7^ cells were collected by centrifugation at 600 × *g* for 5 min at 4 °C and washed with ice-cold PBS. Cells were resuspended with 1 ml of 1 × Cytosol Extraction Buffer Mix containing dithiothreitol and protease inhibitors and incubated on ice for 10 min. Then, cells were homogenized in an ice-cold grinder and the homogenate was transferred to a 1.5 ml microcentrifuge tube and centrifuged at 700 × *g* for 10 min at 4 °C. The supernatant was transferred to a fresh 1.5 ml microcentrifuge tube and centrifuged at 10 000 × *g* for 30 min at 4 °C. The supernatant was collected as cytosolic fraction and the pellet was resuspended in 0.1 ml Mitochondrial Extraction Buffer Mix containing DTT and protease inhibitors and then vortexed for 10 s and saved as mitochondrial fraction.

### Immunoprecipitation

For co-immunoprecipitation of SIRT1, SIRT3, or SOD2 complexes, SIRT1, SIRT3, or SOD2 were immunocaptured from mitochondrial extracts, respectively. Lysate of mitochondrial fraction (1 ml) containing 1.5 mg total protein was incubated with 1 mg SIRT1, SIRT3, or SOD2 antibody, respectively, and 20 ml protein A/G-conjugated beads (Santa Cruz Biotechnology, Santa Cruz, CA, USA) overnight. After four washes in TNES buffer, samples were centrifuged at 3000 × *g* for 2 min and resuspended in 20 ml SDS-sample buffer (0.5M Tris–HCl, pH6.8, 20% glycerol, 2% SDS, 5% 2-mercaptoethanol, 4% bromophenol blue). For western blot analysis, 10 ml samples were used. The immunocomplexes were analyzed by western blotting and probed with antibody against anti-HIF1*α*, or acetylated-lysine antibody (Cell Signaling Technology, Danvers, MA, USA), respectively, and incubated with horseradish peroxidase-conjugated secondary antibody (Santa Cruz Biotechnology).

### Western blotting

Proteins were isolated using lysis buffer, incubated in SDS buffer, separated on SDS-polyacrylamide gels, and electroblotted onto PVDF membranes. Immunoreactive protein bands were detected using an Odyssey Scanning System (LI-COR Inc., Superior St., Lincoln, NE, USA). The following antibodies were used for western blotting: HIF1*α*, PHD1, PHD2, PHD3, SOD2, *β*-actin (Santa Cruz Biotechnology) at 1 : 400 dilution; SIRT3, SIRT1, HKII, Hydroxy-HIF-1*α*, FOXO3a (Cell Signaling Technology) at 1 : 800 dilution.

### Real-time PCR analysis

Total cellular RNA was extracted and purified using the TRIzol reagent (Takara, Otsu, Shiga, Japan) following the manufacturer's instructions. One microgram of total RNA was used to transcribe the first strand cDNA with SuperScript II reverse transcriptase (Invitrogen). Real-time PCR was completed on an ABI PRISM Sequence Detector 7500 (PerkinElmer, Branchburg, NJ, USA) using Sequence Detector version 1.7 software (Applied Biosystems, Foster City, CA, USA). SYBR Green PCR Master Mix was purchased from Applied Biosystems. Forward and reverse primers for targeted mRNA (SIRT3, HIF1*α*, HKII, SOD2, and *β*-actin) were designed and purchased from TAKARA Biotechnology Co., Ltd. (Dalian, China). The primer sequences for real-time PCR were listed as below:

SIRT3: (sense) 5′-CATTAAATGTGGTGGAACAAGAGGCCTG-3′

SIRT3: (antisense) 5′-AGTTCCTCTCCTTTGTAATCCCTCCGAC-3′.

SOD2: (sense) 5′-GCACATTAACGCGCAGATCA-3′

SOD2: (antisense) 5′-AGCCTCCAGCAACTCTCCTT-3′.

HIF1*α*: (sense) 5′-CAGCCGCTGGAGACACAATC-3′

HIF1*α*: (antisense) 5′-TTTCAGCGGTGGGTAATGGA-3′.

HKII: (antisense) 5′-CAAAGTGACAGTGGGTGTGG-3′

HKII:(antisense) 5′-GCCAGGTCCTTCACTGTCTC-3′.

*β*-actin: (sense) 5′-CTGTCCCTGTATGCCTCTG-3′

*β*-actin: (antisense) 5′-ATGTCACGCACGAT-TTCC-3′.

Fold change of mRNA level was calculated as follows. After completion of the PCR, the baselines and thresholds were set for both samples and internal *β* -actin controls. Using Ct values (cycle number in which the sample crosses the threshold value) for samples (sam) and controls, the ΔCt was calculated as follows: ΔCt=Ct_sam_ - Ct_actin_. The values for each sample were then compared with those of the control sample (ctl): ΔΔCt =ΔCt _sam_ -ΔCt _ctl_. The fold change of the mRNA level to the control is 2^-ΔΔCt^.

### SOD2 activity assay

SOD2 activity was assayed with the Cu/Zn-SOD and Mn-SOD Assay Kit (KeyGen, Nanjing, China) following the manufacturer's instructions. Breifly, cells were collected and lysed. The addition of 3 mM potassium cyanide to the cell lysate inhibited both Cu/Zn-SOD and extracellular SOD, resulting in the detection of only Mn-SOD activity. Samples were assayed in the absence of xanthine oxidase to generate a sample background. After sample and SOD standard were prepared and added into 96-well plate, we initiated the reaction by adding 20 *μ*l of diluted xanthine oxidase to all the wells. The plate was incubated on a shaker for 30 min at room temperature. The OD values were detected using a spectrophotometer (Thermo) at 450 nm.

The SOD activity of the sample was calculated using the equation obtained from the linear regression of the standard curve substituting the linearized rate (LR, LR= (A_blank1_-A_blank2_-A_sample_) / (A_blank1_-A_blank2_) × 100%) for each sample. One unit is defined as the amount of enzyme needed to exhibit 50% dismutation of the superoxide radical. Thus, SOD activity _sample_(U) =LR _sample_ /(1-LR _sample_) units.

### Electrophoretic mobility shift assay (EMSA)

An EMSA was performed using the Chemiluminescent EMSA Kit (Beyotime, Nantong, China). After the nuclear proteins were obtained, the EMSA was performed according to the instructions of the manufacturer.

The probe for the FOXO3a DNA binding region (SOD2 promoter) was 5′-cAGGCTGGGCGGCGGga**gctc**acgcgtCCGCGAAGAAACgct**agcc**tcgagCTCCTGGCTTTa-3′ (Jacobs indicates FOXO3a binding sites^31^). The mutant probe was 5′- cAGGCTGGGCGGCGGga**gttt**acgcgtCCGCGAAGAAACgct**aggg**tcgagCCTCCTGGCTTTa-3′.

### ChIP assay

A ChIP assay was performed using the ChIP Assay Kit (Beyotime). MDA-MB-231 cells were treated with OA under hypoxia for 10 h. Cells were then cross-linked with formaldehyde, quenched with glycine, sonicated on ice, and centrifuged at 4 °C. Mixtures were incubated with FOXO3a (Cell Signaling Technology) or preimmune IgG with rotation at 4 °C overnight and then incubated with protein A***/***G agarose at 4 °C for 6 h. Finally, immune complexes were captured by protein A***/***G agarose and eluted with elution buffer (1% SDS and 0.1M NaHCO3) at 37 °C for 30 min. Cross-linking was reversed at 65 °C for 4 h in a high salt buffer (0.2 M NaCl, 50 mM Tris, pH 6.5, 10 mM EDTA, and 0.2 mg/ml proteinase K). Extracted and dissolved immunoprecipitated DNA was quantified by real-time PCR with primers encompassing the Foxo3a-binding sites. The primers used for real-time PCR to quantitate the ChIP-enriched DNA for SOD2 were as follows: 5′-TCTGACGTCTGTAAACAAGCCCAG-3′ (forward) and 5′-TTCTTTCCTGCGCTGT-CTTGTAGC-3′ (reverse). An equal volume of nonprecipitated (input) genomic DNA was used to correct for the differences in PCR amplification efficiencies and amounts of DNA. The PCR analyses were performed with a real-time PCR kit (TaKaRa Biotechnology Co., Ltd).

### Cell transfection and luciferase reporter assay

For transient transfection, MDA-MB-231 cells (5 × 10^5^ cells per well) were plated in 6-well plates. Cells were co-transfected with 0.4 *μ*g per well Renilla luciferase reporter and 4 *μ*g per well p3x-FOXO3a-luc plasmid containing FOXO3a-binding motifs (cAGGCTGGGCGGCGGgagctcacgcgtCCGCGAAGAAACgctagcctcgagCCTCCTGGCTTTa, Beyotime). Then, the cells were treated for 10 h by OA. Luciferase assay was performed with the Luciferase Reporter Gene Assay kit (Promega, Madison, WI, USA) and signals were detected using Luminoskan ascent (Thermo).

### *In vivo* tumor growth assay

Female athymic BALB/c nude mice (35–40 days old) with body weight ranging from 18 to 22 g were supplied by the Academy of Military Medical Sciences of the Chinese People's Liberation Army (Certificate No. SCXK-(Army) 2007-004). The animals were kept at 22±2 °C and 55−65% humidity in stainless steel cages under controlled lights (12 h light/day) and were fed with standard laboratory food and water. Animal care was conducted in accordance with the recommendations of the Guide for the Care and Use of Laboratory Animals published by the National Institute of Health, USA.

This experiment was conducted in accordance with the guidelines issued by the State Food and Drug Administration (SFDA of China).Forty nude mice were inoculated subcutaneously with 1 × 10^7^ MDA-MB-231 cells into the right axilla. After 12 days of growth, tumor sizes were determined using micrometer calipers. Mice with similar tumor volumes (mice with tumors that were too large or too small were eliminated) were randomly divided into the following five groups (6 mice per group): saline control, OA (50 mg/kg, i.v., every 2 days ), OA (100 mg/kg, i.v., every 2 days), 2-DG (500 mg/kg, i.p., every 2 days), and ADM (4 mg/kg, i.v., twice a week). Tumor sizes were measured every 3 days using micrometer calipers and tumor volume was calculated using the following formula: TV (mm^3^)=d^2^ × D/2, where d and D were the shortest and the longest diameter, respectively. Mice were killed on day 21, and tumor tissues were used for western blotting and immunohistochemistry assay.

And the Inhibitory rate (%) of tumor growth was calculated as below:


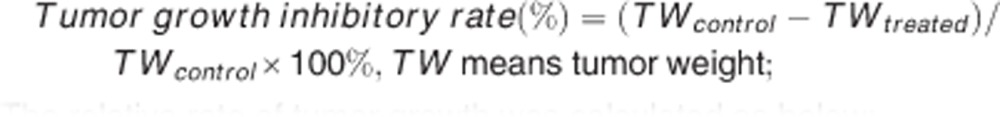


The relative rate of tumor growth was calculated as below:


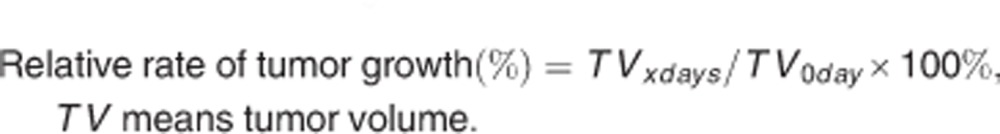


### Immunohistochemistry

The expression of HIF-1*α*, HKII, SIRT3, and SOD2 in the tissues of the control and treated (OA 50 mg/kg, 100 mg/kg, 2-DG 500 mg/kg, and ADM 4 mg/kg) groups was assessed by the SP immunohistochemical method using a rabbit-antihuman monoclonal antibody and an Ultra-Sensitive SP kit (kit 9710 MAIXIN, Maixin-Bio Co., Fujian, China). Tissue sections (4-mm thick) were placed onto treated slides (Vectabond, Vector Laboratories, Burlingame, CA, USA). Sections were heat-fixed, deparaffinized, and rehydrated through a graded alcohol series (100, 95, 85, 75%) to distilled water. Tissue sections were boiled in citrate buffer at high temperature for antigen retrieval, and treated with 3% hydrogen peroxide to block endogenous peroxidase activity. The slides were incubated with a protein-blocking agent (kit 9710 MAIXIN, Maixin-Bio Co., Fuzhou, Fujian) prior to the application of the primary antibody, and then incubated with the primary antibody at 4 °C overnight. The tissues were then incubated with the secondary biotinylated antispecies antibody and labeled using a modification of the avidin–biotin complex immunoperoxidase staining procedure according to the Ultra-Sensitive SP kit (ZSGB-BIO Inc., Beijing, China). Counterstaining was carried out using Harris hematoxylin.

### siRNA-mediated knockdown of sirtuin-3 or SOD2

The siRNAs targeting SIRT-3 or SOD2 were delivered by a lipid-based method using Lipofectamine 2000 (Invitrogen Life Technologies, Grand Island, NY, USA) at a final siRNA concentration of 30 *μ*M. After formation of the siRNA–liposome complexes, the mixture was added to breast cancer cells for 4 h. The medium was then aspirated and replaced with complete medium containing 100 *μ*M OA.

### Transfection of HIF1*α* and SIRT3 plasmids

The HIF1*α* human cDNA clone and SIRT3 human cDNA clone were obtained from OriGene (OriGene Technologies, Inc., Rockville, MD, USA). For transfection, cells were seeded in 6-well plates at 65% confluency at first. Then, the plasmid DNA (1 *μ*g) was introduced into the cells using Lipofectamine 2000 (Invitrogen Life Technologies) according to the manufacturer's recommendations. Cells were then exposed to OA or the vehicle and harvested for further experiments.

### Statistical evaluation

Data are presented as mean±S.D. from triplicate parallel experiments unless otherwise indicated. Statistical analyses were performed using one-way ANOVA. Least Significant Difference test and Tukey's HSD test were used for the one-way ANOVA analyses.

## Abbreviations

SIRT3: sirtuin 3
SOD2: superoxide dismutase
HIF1*α*: hypoxia-inducible factor 1*α*
GLUT: glucose transporter 1
HKII: hexokinase II
PDK1: pyruvate dehydrogenase kinase 1
ROS: reactive oxygen species
PHDs: prolyl hydroxylases
FOXO3a: forkhead box O3a
DMOG: dimethyloxaloylglycine
TSA: trichostatin A
NAM: nicotinamide
ADM: Adriamycin
mtDNA: mitochondrial DNA
Ac-SOD2: acetylated-SOD2

## Notes

The authors declare no conflict of in interest.
