# Supplementary figures and images for: Oroxylin A inhibits glycolysis-dependent proliferation of human breast cancer via promoting SIRT3-mediated SOD2 transcription and HIF1α destabilization
Source: Cell Death Dis. 2015 Apr 9;6(4):e1714–. doi: 10.1038/cddis.2015.86 (PMC4650553; doi:10.1038/cddis.2015.86)

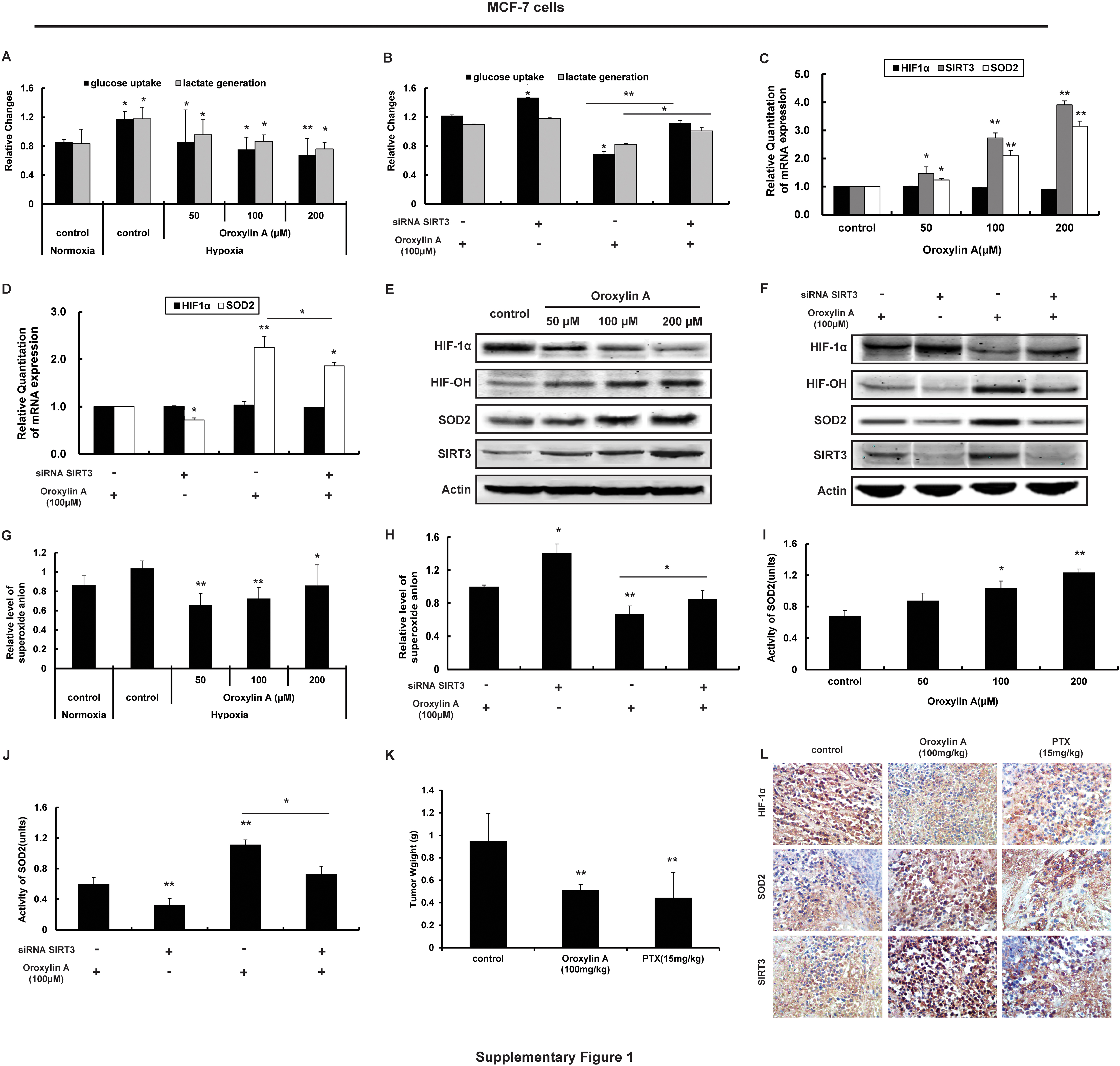

Supplement: Supplementary Figure 1 [file cddis201586x1.tif]

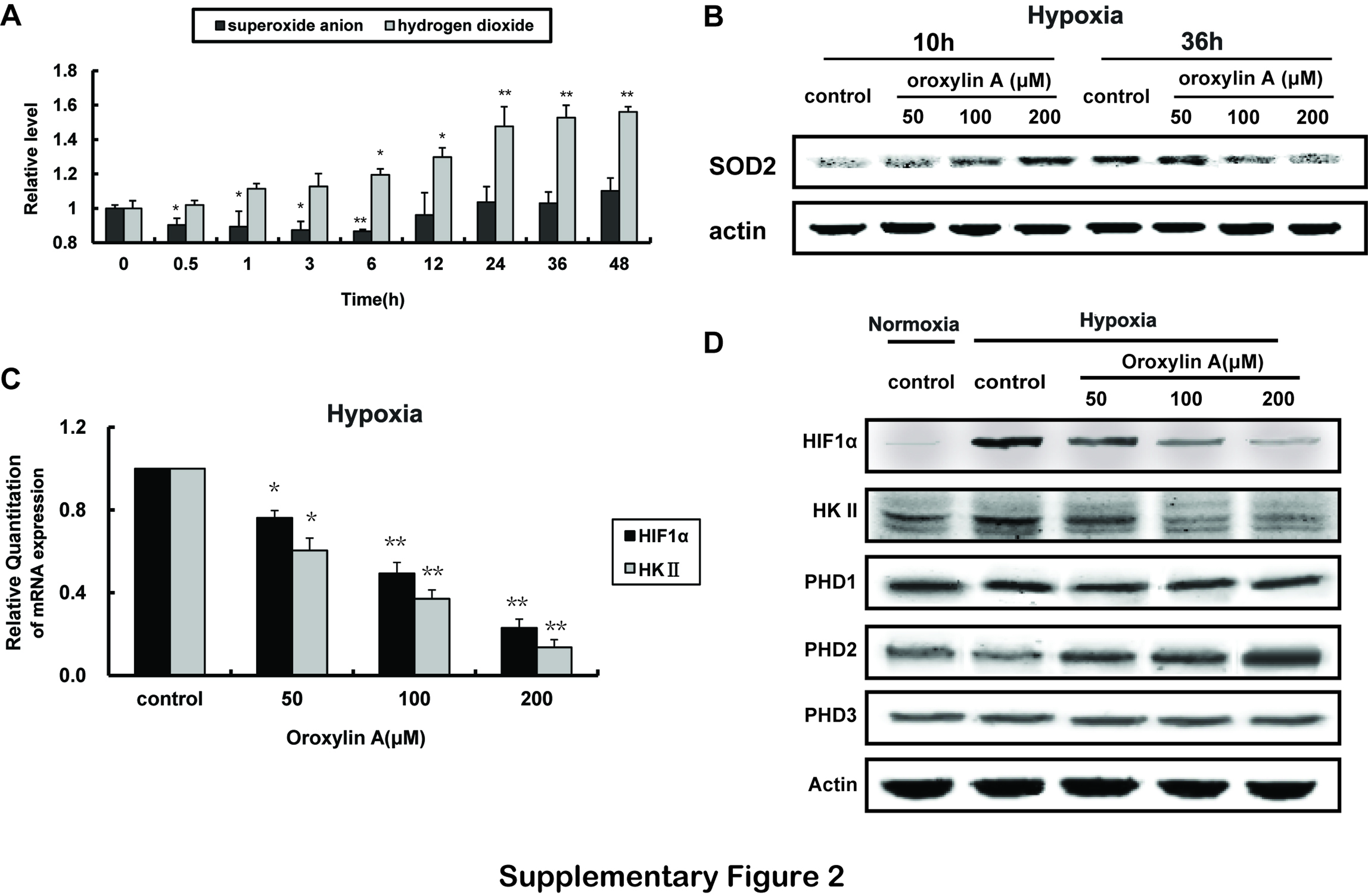

Supplement: Supplementary Figure 2 [file cddis201586x2.tif]

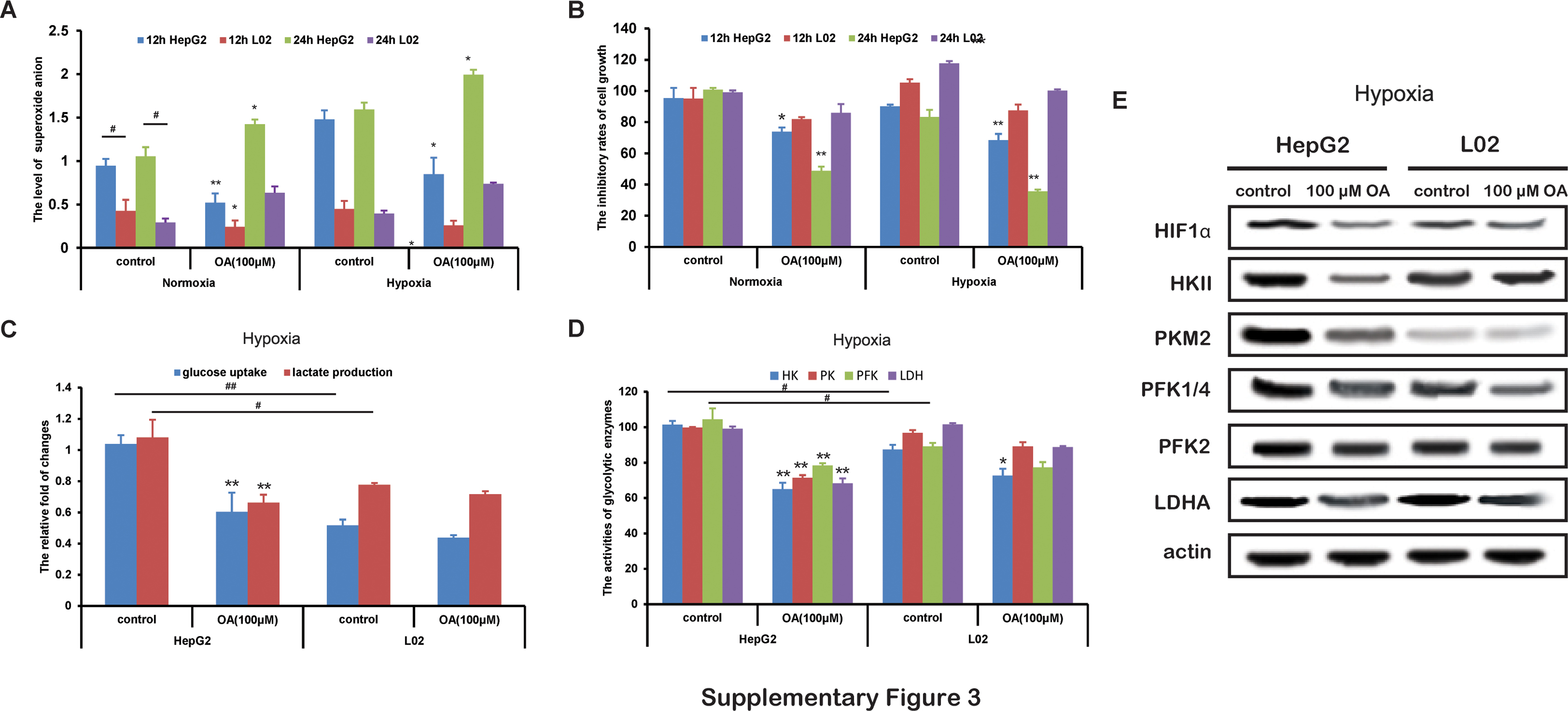

Supplement: Supplementary Figure 3 [file cddis201586x3.tif]

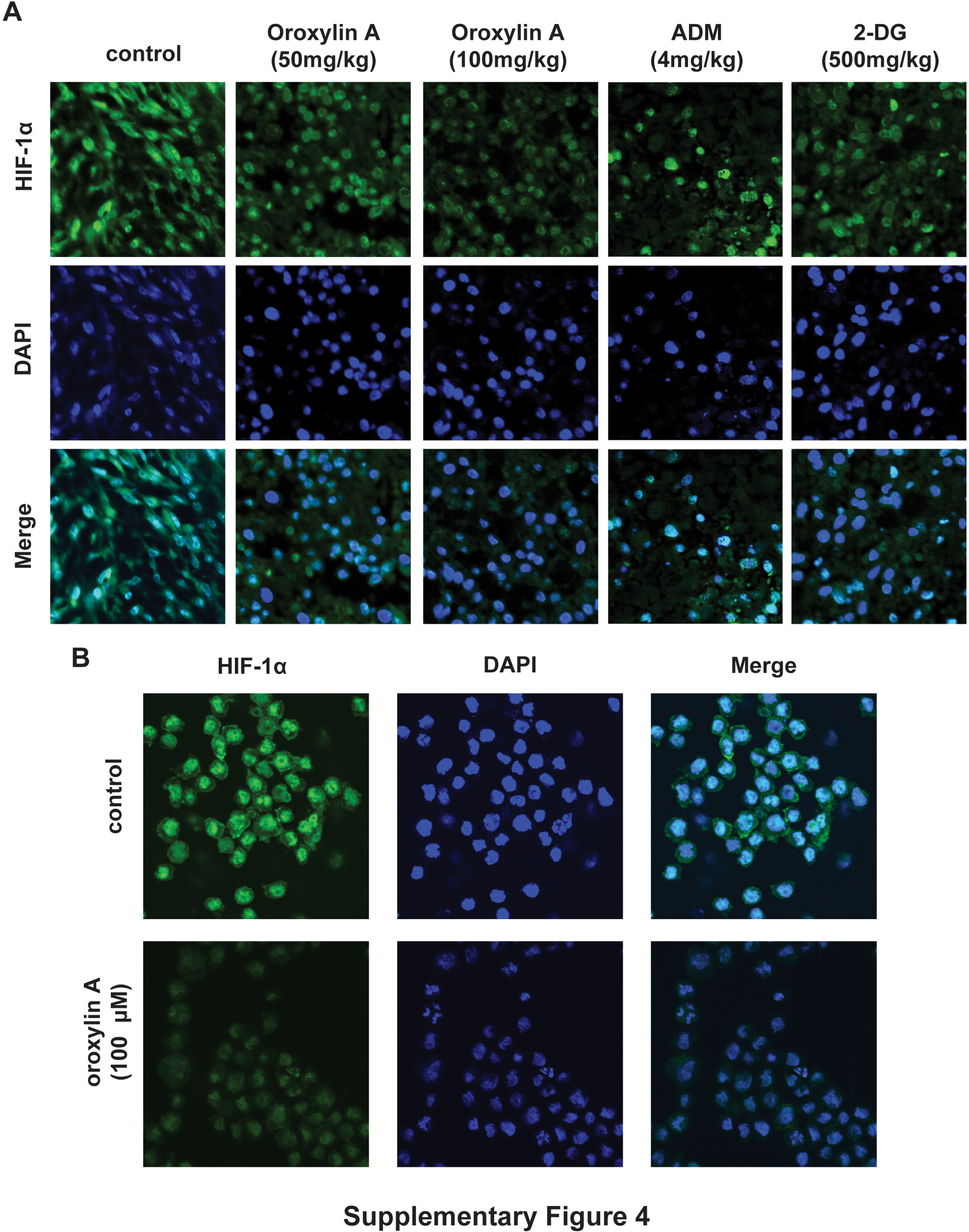

Supplement: Supplementary Figure 4 [file cddis201586x4.tif]

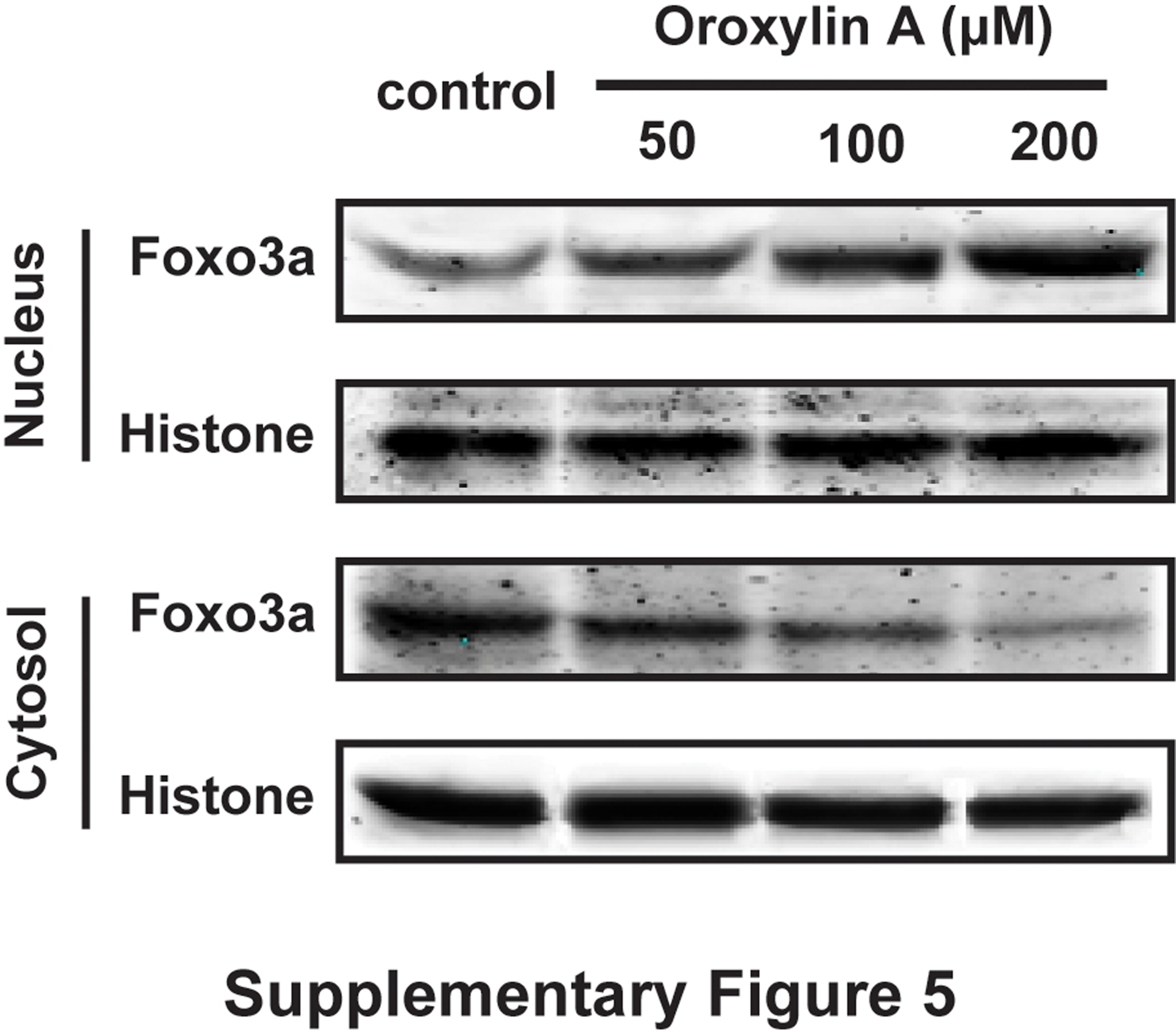

Supplement: Supplementary Figure 5 [file cddis201586x5.tif]

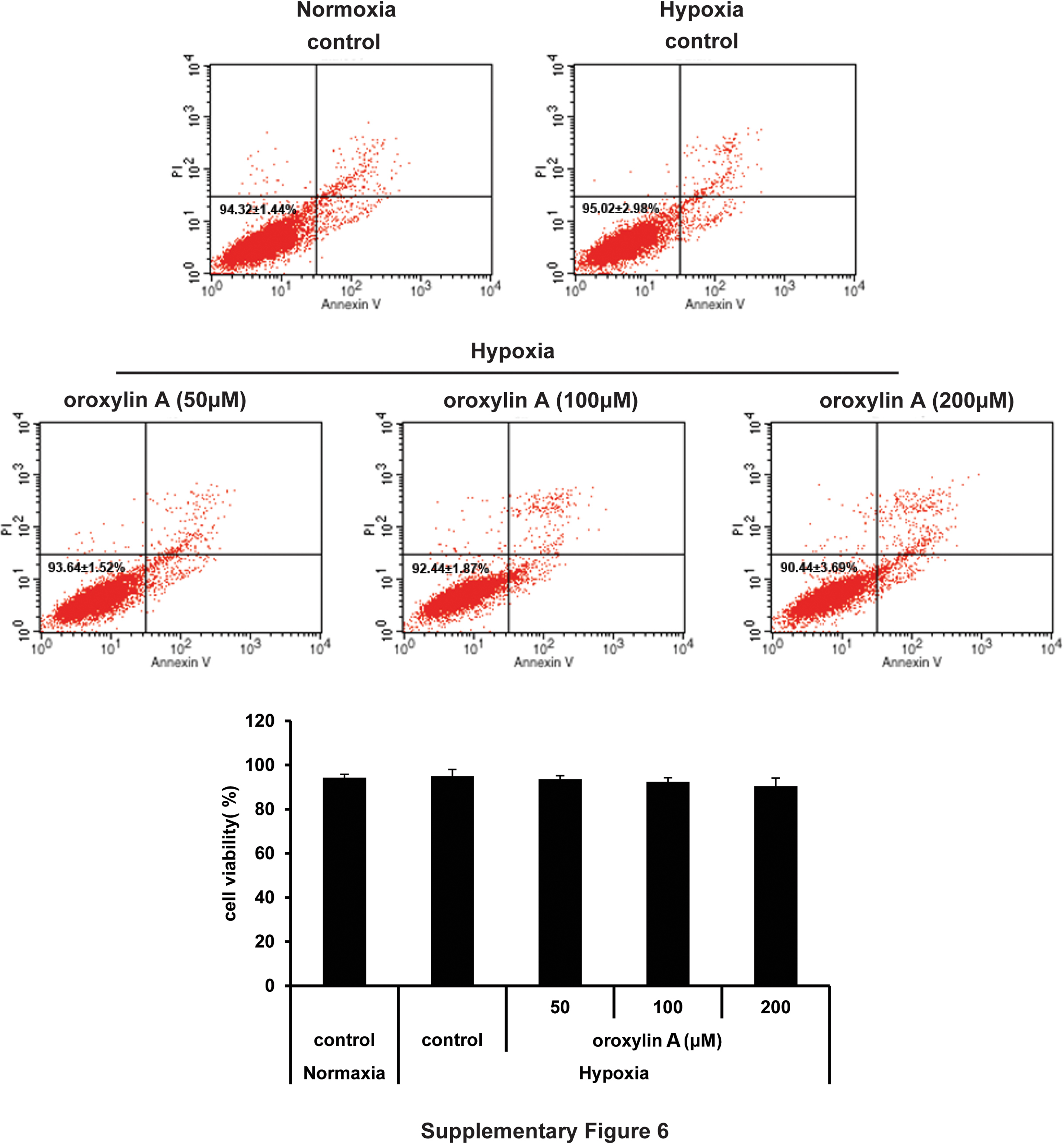

Supplement: Supplementary Figure 6 [file cddis201586x6.tif]
