# Supplementary material for: Oroxylin A inhibits glycolysis-dependent proliferation of human breast cancer via promoting SIRT3-mediated SOD2 transcription and HIF1α destabilization
Source: Cell Death Dis. 2015 Apr 9;6(4):e1714–. doi: 10.1038/cddis.2015.86 (PMC4650553; doi:10.1038/cddis.2015.86)
Supplement: Supplementary Figure Legends [file cddis201586x7.doc]

**Supplementary Figure Legends**

**Supplementary Figure 1 The influence of oroxylin A on the glycolysis and the stability of HIF1α in MCF-7 cells under hypoxia.** (A, C, E, G, I) MCF-7 cells were treated with oroxylin A under conditions of hypoxia or normoxia for 10 h. (B, D, F, H, G) SIRT3-deficient MVF-7 cells were incubated with/without 100 μM oroxylin A for 10 h under hypoxia. (A, B) Glucose uptake was measured using the Amplex Red assay, and production of lactic acid was assayed with the Lactic Acid production Detection kit. (C, D) The mRNA expressions of SIRT3, HIF1α, and SOD2 II were detected by quantitative RT-PCR. (E, F) The protein expressions of SIRT3, HIF1α, hydroxylated HIF1α (HIF-OH) and SOD2 II were detected by immunoblotting. (G, H) The levels of cellular superoxide anion (•O_2_^−^) was detected by FACSCalibur flow cytometry using the fluorescent dye DHE at Ex/Em of 300/610 nm. (I, J) SOD2 was detected by Cu/Zn-SOD and Mn-SOD Assay Kit. (K, L) Nude mice inoculated with MCF-7 cells were treated with saline control, oroxylin A, and paclitaxel (PTX). (K) The tumor inhibitory rates were calculated. (L) Proteins expressions in breast tumor tissue were assessed by immunohistochemistry. Bars, SD; *p<0.05 or **p<0.01 versus untreated controls in hypoxia.

**Supplementary Figure 2 The long-term efficacy of oroxylin A on the cellular ROS level and HIF1α stability under hypoxia.** (A) MDA-MB-231 cells were treated with oroxylin A under hypoxia. The levels of •O_2_^−^ and H_2_O_2_ were detected by FACSCalibur flow cytometry, respectively. (B) The protein expression of SOD2 in oroxylin A-treated MDA-MB-231 cells for 10 h and 36 h were detected by immunoblotting. (C) The mRNA expressions of HIF1α and HK II in the oroxylin A-treated cells for 36 h under hypoxia were detected by quantitative realtime-PCR. (D) The protein expressions of HIF1α, HKII, and PHDs (PHD1, PHD2, PHD3) were detected by immunoblotting. Bars, SD; *p<0.05 or **p<0.01 versus untreated control.

**Supplementary Figure 3 The different effects of oroxylin A on cell growth and glycolysis in hepatoma cells and normal hepatic cells.** HepG2 cells and L02 cells were treated with 100 μM oroxylin A for 12 h or 24 h under normoxia or hypoxia. (A) The levels of cellular superoxide anion (•O_2_^−^) were detected by FACSCalibur flow cytometry (B) The cell growth rate of oroxylin A was measured by MTT assay. (C) Glucose uptake was measured using the Amplex Red assay, and production of lactic acid was assayed by Lactic Acid production Detection kit. (D) The activities of the key glycolytic enzymes. The activities of HK, PFK, PK and LDH were detected by the corresponding enzyme activity assay kit. (E) The protein expressions of HIF1α and some key glycolytic enzymes. Bars, SD; **p*<0.05 or ***p*<0.01 versus corresponding untreated control with the same treatment time and conditions. ^#^*p*<0.05 or ^##^*p*<0.01 indicate significant differences between the two items linked.

**Supplementary Figure 4 The influence of oroxylin A on the subcellular localization of HIF1α.** (A) Xenograft model of MDA-MB-231 cells was constructed. The expression and distribution of HIF1α in the tumor tissue was detected by Immunofluorescence. (B)Immunofluorescence experiment performed in MDA-MB-231 cells upon oroxylin A treatment using antibodies specific to HIF1α and DAPI.

**Supplementary Figure 5 The influence of oroxylin A on the nuclear translocation of Foxo3a.** Nucleus and cytosolic fractions were isolated after treatment and subjected to western blot analysis for Foxo3a.

**Supplementary Figure 6 The role of oroxylin A on cell survival.** Annexin-V/PI double-staining assay were measured after cells were treated with oroxylin A under hypoxia conditions for 10 h. Histograms of living cells rates were quantitated. Bars, SD.
