# Supplementary material for: Oroxylin A inhibits glycolysis-dependent proliferation of human breast cancer via promoting SIRT3-mediated SOD2 transcription and HIF1α destabilization
Source: Cell Death Dis. 2015 Apr 9;6(4):e1714–. doi: 10.1038/cddis.2015.86 (PMC4650553; doi:10.1038/cddis.2015.86)
Supplement: Supplementary Information [file cddis201586x8.doc]

**Supplementary Materials & Methods**

***Cell Culture***

The human breast cancer cell line MCF-7 cell, the human hepatoma cell line HepG2 cell, and human normal hepatic cell line L02 cell were purchased from Cell Bank of Shanghai Institute of Biochemistry & Cell Biology, Chinese Academy of Sciences (Shanghai, China). MCF-7 cells was cultured in and Dulbecco's MEM (DMEM, Invitrogen Corporation, Carlsbad, CA); HepG2 cell and L02 cell were cultured in RPMI Media 1640 Medium (GIBCO, Invitrogen Corporation, Carlsbad, CA), both supplemented with 10% heat-inactivated fetal bovine serum (Sijiqing, Hangzhou, China), 100 U/ml penicillin G , and 100 μg/ml streptomycin at 37°C, 95% relative humidity, and 5% CO_2_ with and 5% CO_2_ with 20% oxygen (normoxia), or 1% oxygen (hypoxia).

***In vivo tumor growth assay of the nude mice inoculated with MCF-7 cells***

To facilitate estrogen-dependent xenograft establishment, each mouse received 17-estradiol (20 mg/kg; Sigma) intraperitoneally once a week. One week after treatment, equivalent amounts of MCF-7 cells were injected subcutaneously (10^7^ cells/tumor) into the left axilla of nude mice. After 12 days of growth, tumor sizes were determined using micrometer calipers. Mice inoculated MCF-7 cells with similar tumor volumes were randomly divided into the following 3 groups (6 mice/group): saline control, oroxylin A (100 mg/kg, i.v., every 2 days ), and paclitaxel (PTX, 15 mg/kg, i.v. twice a week)

Tumor sizes were measured every 3 days using micrometer calipers and tumor volume was calculated using the following formula: TV (mm3) = d^2^×D/2, where d and D were the shortest and the longest diameter, respectively. Mice were sacrificed on Day 21, and tumor tissues were used for Western blotting and Immunohistochemistry Assay.

***Spectrophotometric assay for enzymes activity***

Cells were seeded in 24-well plates and grown to confluence. Then, medium was removed and fresh medium was added, and cells were returned to the incubator in the presence of 100 μM oroxylin A for 12 h or 24 h under normoxia or hypoxia, respectively. After this incubation, cells were removed from the plates by trypsinization and counted using a hemocytometer. Protein concentrations of cell lysates were measured, and the glycolytic enzyme activities were assayed.

Cellular HK activity, PK activity, PFK activity and LDH activity were respectively measured by colorimetric assay, following the instructions of Hexokinase Activity Assay Kit, Pyruvate kinase activity Assay Kit, Phosphofructokinase activity Assay Kit and Lactate Dehydrogenase Activity Assay Kit (BioVision, Inc., Milpitas, CA).

HK activity assay: after NADH Standard and samples were prepared in the 96-wells plate, incubate the plate 20-60 min at 37°C and measure OD at 450nm by spectrophotometer (Thermo, Waltham, MA, USA). Calculate the hexokinase activity of the test sample: ΔOD = A2 – A1 (A1 was the OD in 0 min, and A2 was the OD in 5 min). Apply the ΔOD to the NADH standard curve to get B nmol of NADH generated by hexokinase during the reaction time (ΔT = T2 - T1).

Sample Hexokinase activity = B/(ΔT X V) x Dilution Factor = nmol/min/ml/ = mU/ml. (Where: B is the NADH amount from standard curve (nmol).ΔT is the reaction time (min). V is the sample volume added into the reaction well (ml).)

b) PK activity assay.

The assay methods of PK activity, PFK activity and LDH activity assay were generally as same as HK activity assay with a little adjustment. In LDH activity assay, Pyruvate Standard was used and samples OD were measure at 570 nm.

***Immunofluorescence and confocal fluorescence microscopy***

Cells were fixed with 4% paraformaldehyde in PBS at 1 h intervals, permeabilized with 0.5% Triton X-100, and blocked with 3% BSA for 30 min. Tissue sections were heat fixed, deparaffinized and rehydrated through a graded alcohol series (100%, 95%, 85%, 75%) to distilled water. Tissue sections were boiled in citrate buffer at high temperature for antigen retrieval, and treated with 3% hydrogen peroxide to block endogenous peroxidase activity. The slides were incubated with a protein-blocking agent (kit 9710 MAIXIN, Maixin-Bio Co., Fujian, China) prior to the application of the primary antibody.

Incubation with primary antibodies against HIF1α was done overnight at 4°C. Then the nuclei were stained with 4’,6-diamidino-2-phenylindole (DAPI, Sigma-Aldrich) 20 min before imaging. A laser scanning confocal microscope FV10-ASW (Ver 2.1) (Olympus Corp, MPE FV1000, Tokyo, Japan) was used for colocalization analysis.

***Preparation of nuclear- and cytosol-enriched extracts***

After cells were incubated with oroxylin A for 10 h under hypoxia, cell nuclear and cytoplasmic fractions were prepared using a nuclear/cytosol fractionation kit of Biovision Inc. (Mountain View, CA, USA) according to the manufacture’s direction.

***Apoptosis assay***

Cells were incubated with oroxylin A for 10 h in hypoxia, then harvested and resuspended with PBS. Apoptotic cells were identified by double supravital staining with recombinant FITC (fluorescein isothiocyanate)-conjugated Annexin-V and PI, according to the manufacturer’s instructions of the Annexin-V-FITC Apoptosis Detection kit (KeyGen, Nanjing, China). Apoptotic cell death was examined by FACSCalibur flow cytometry (Becton Dickinson, San Jose, CA).
